# Evaluation of NETosis (Neutrophil Extracellular Traps) biomarkers in smokers and nonsmokers periodontitis patients

**DOI:** 10.12688/f1000research.152917.2

**Published:** 2025-04-07

**Authors:** Rasha Abdul Jabbar Najim, Batool Hassan Al Ghurabi

**Affiliations:** 1 Master's student, Basic Science Department, University of Baghdad, Baghdad, Baghdad Governorate, Iraq; 2 Microbiological/Immunological Professor, Basic Science Department, University of Baghdad, Baghdad, Baghdad Governorate, Iraq

**Keywords:** Biomarkers; Calprotectin; Citrullinated histone H3; Myeloperoxidase; NETosis; Neutrophil elastase; Periodontitis

## Abstract

**Background:**

To evaluate the NETosis biomarkers citrullinated histone H3 (citH3), neutrophil elastase (ELA), calprotectin (CALPRO), and myeloperoxidase (MPO) as indicators of inflammation in the severe stages of periodontitis III and IV in both (smokers and nonsmokers) patients, and to determine the correlation between NETosis biomarkers and clinical periodontal parameters.

**Methods:**

This study recruited male subjects with an age range of (20-60) years; 60 were stage III and stage IV periodontitis patients, 30 were cigarette smoker, and 30 were nonsmokers. After applying the inclusion and exclusion criteria to evaluate their eligibility for recruitment, 25 control subjects with a healthy periodontal status and good oral hygiene maintenance were included. Unstimulated saliva was obtained and evaluated using an enzyme-linked immunosorbent assay, and the following periodontal parameters were documented: [plaque index, bleeding on probing, periodontal pocket depth, and clinical attachment loss].

**Results:**

The mean levels of all salivary NETosis biomarkers citH3, ELA, CALPRO, and MPO were elevated in the periodontitis groups (smokers and nonsmokers) than in controls. Moreover, the mean NETosis biomarker‘s mean levels were significantly higher in smoker than in nonsmokers. In addition, the correlations were significant between CALPRO and CitH3 in smokers and between ELA and CitH3 in nonsmokers.

**Conclusions:**

The results of this study showed that the chosen salivary biomarkers of NETosis revealed elevated clinical accuracy in differentiating the studied periodontitis groups (smokers and nonsmokers) from controls. In addition, cigarette smoking increases the risk of periodontitis, and neutrophils in smokers with periodontitis exhibited more susceptibility to form neutrophil extracellular traps when compared with nonsmokers.

**Abbreviations**
BOP: Bleeding On Probing
citH3: Citrullinated histone H3
CAL: Clinical Attachment Loss
CALPRO: Calprotectin
ELISA: Enzyme-Linked Immunosorbent Assay
MPO: Myeloperoxidase
NETs: Neutrophil Extracellular Traps
PLI: Plaque index
PPD: Periodontal Pocket Depth

## Introduction

Periodontitis is a multifactorial chronic, irreversible inflammation; however, the primary cause of periodontitis has been found to be the bacterial biofilm that develops on tooth surfaces, the host’s reaction, in addition to local elements such as plaque, calculus, external factors, and the health of the patients in general, all of which influence disease progression. ^1^ Smoking is one of the main ways in which individuals are exposed to harmful chemicals (cigarette smoke contains at least 4,500 different chemicals), and can cause epigenetic changes that increase inflammation. In addition, smoking generally increases oxidative stress, with excess free oxygen radicals causing cell damage. As a result, smoking exacerbates periodontal disease and negatively affects nearly all forms of periodontal therapy. ^2^^,^^3^ Several studies have indicated that smokers are more likely to have periodontal disease than nonsmokers, including greater gingival recession, bone attachment, and tooth loss. ^4^^–^^7^ Reactive oxygen species (ROS) released by periodontal infections and their byproducts activate several phagocytes, including neutrophils, monocytes, gingival fibroblasts, and cells found in the periodontal tissue. In patients with periodontitis, there is an increase in the recruitment of polymorph nuclear neutrophils (PMNs), which results in the breakdown of bone and oral tissues, ^1^^,^^8^^–^^10^ this is because the periodontium is a special habitat where oral bacteria are constantly in contact with the host immune system. Neutrophils serve as the initial line of defense of the human innate immune system and have the ability to eliminate infections extracellularly, connect innate and adaptive immune responses, and support tissue healing and inflammatory resolution. ^9^^,^^11^^,^^12^ However, severe periodontitis is caused by a lack of neutrophils, and an excess of neutrophils appears to be harmful because it maintains the body in an inflammatory state, which destroys tissue. Maintaining a balance between neutrophils and other macrophages is crucial for maintaining periodontal homeostasis, as an imbalance can result in periodontitis. ^8^^,^^13^ According to these recent studies, ^3^^,^^14^ smoking cigarettes has several potential detrimental effects on periodontal health, including a decrease in wound healing time, vascular constriction, impaired defenses against plaque bacteria, and interference with oxidative stress processes. Because they disturb redox equilibrium, reactive oxygen species (ROS) are toxic to humans, and exposure to cigarette smoke increases their levels. However, sex hormones may also affect the gingival microcirculation, which can affect the global spread of the disease. Men are substantially more likely than women to show vasodilation in case of inflammation or when a wound is healing and when the disease is actively progressing. ^3^^,^^15^^–^^19^ Neutrophil equilibrium is important in periodontal homeostasis because periodontitis can develop when there are too many or too few neutrophils in the tissue. ^8^ One means by which Neutrophils aid in homeostasis and defense by producing neutrophil extracellular taps (NETs), which allow them to capture bacteria in the extracellular space by releasing decondensed chromatin adorned with histones and granule content. NETosis is defined as the occurrence of neutrophil death and NET production. The primary defense of periodontal tissues is provided by NETs, and a deficiency in NETs can result in severe early-onset periodontitis. ^9^^,^^18^^,^^19^ Smoking cigarettes accelerates the production of NETs and PMN-derived proteases, which are responsible for breaking down connective tissue. ^2^^,^^14^ NETs are composed of DNA and histones, along with associated neutrophil granule proteins such as citH3, Neutrophil Elastase, Calprotectin and Myeloperoxidase. ^12^ Histone H3 is the most abundant protein in NETs, and citrullination of histones is a specific marker of NETosis. ^20^^,^^21^ ELA is a serine protease maintained in abundance in azurophilic granules of neutrophils, together with other proteins involved in anti-microbial defense, and high levels of ELA in saliva can be associated with the loss of active periodontal attachment. ^22^ CALPRO belongs to the S100 family, which accounts for 5–40% of the cytosolic protein content of neutrophils. It acts as a new warning system by demonstrating pro-inflammatory activities primarily induced by activated granulocytes in a calcium-dependent manner. It contributes by binding to specific cell surface receptors, which sets off further signal transductions that contribute to the recruitment of white blood cells and production of inflammatory cytokines in the affected regions. ^23^ One of the many mediators released in tissues as inflammation advances is MPO, whose expression is highly correlated with gingival tissue in periodontitis. ^24^ Based on the available evidence, this study evaluated the NETosis biomarkers as indicators of inflammation in stage III and stage IV (smokers and nonsmokers) periodontitis groups and compared the findings with those of the controls. Furthermore, we determined the relationship between the NETosis biomarkers and clinical periodontal parameters.

## Methods

### Study design

This was a case-control study based on observational data. The study was carried out in the Periodontics Department/College Dentistry, University of Baghdad, Al-Baladiat specialized center for dentistry, Al Sader specialized center for dentistry, and Nawar Mousa specialized center for dentistry in Baghdad/Iraq, from October 18, 2023, to January 2024. Ethical considerations were essential during the conduct of this study, introduced by the World Medical Association’s Helsinki Declaration (WMA), after submission to the ethical committee at the Dentistry College/University of Baghdad to obtain ethical approval (Reference Number: 844, project number: 844823, date: 15-10-2023).

### Study population

The 85 male subjects recruited in the study and selected according to their oral and periodontal health status, the type of this study was case control study not clinical trial study, Depending on the classification criteria by Tonetti, ^25^ the selected patients were classified into:

• Periodontal healthy control group with intact periodontium.
• Stage III periodontitis (loss of bone reaches the root’s midpoint).
• Stage IV periodontitis (loss of bone reaches the apical third of the root). Each periodontitis group was further subcategorized into two groups:
• Smokers (stage III and stage IV).
• Non-smokers (stage III and stage IV).

### Inclusion criteria

All males recruited in the study had at least 20 teeth and no systemic disease with no antibiotic consumption for the last 3 months. The control group with healthy periodontium exhibiting good oral hygiene without any periodontal disease history or symptoms with intact periodontium (BOP<10% and PPD≤3), ^26^ On the other hand, cases of periodontitis were chosen based on the following criteria, ^25^ which defined the disease as having visible interdental CAL at two or more teeth that are not adjacent, CAL measuring more than 3 mm along with pocketing measuring at least 3 mm, can be found at two or more teeth, stage III and stage IV, in which the percentage of the loss of bone reaches the midpoint and apical third of roots, as well as unstable with the depth of the pocket of more than 5 mm or when bleeding occurs upon probing at a depth of 4 mm.

### Exclusion criteria

These criteria included subjects with the following: systemic diseases such as autoimmune diseases and diabetes mellitus, history of smoking or alcohol drinking, use of anti-inflammatory drugs and/or antibiotics within the previous three months, use of any periodontal treatment that was used in the past or at present, orthodontic appliances or prosthodontics, consumption of any type of smoke other than cigarettes (electric cigarettes and vapes), oral lesions such as lichen planus, and other diseases unrelated to periodontitis; Females excluded from the study.

### Sample size

The G power 3.1.9.7 program (developed by Franz-Faull, Kiel University/Germany) was employed to compute the size of the sample. Under these conditions, the sample size is around 85 participants, with a power of 90%, an alpha error of 0.05 on both sides’ results in, a significant effect size of F at 0.4, and three groups. ^27^

Effect size F (Small =0.1, Medium=0.25, Large=0.4).

### Saliva sample collection

The patients were advised to clean and floss their teeth to maintain good oral hygiene. Unstimulated saliva was collected prior to clinical examination. Salivary samples were conducted between 9 am and 12 pm. The participants’ saliva was drooled passively into a plastic cup without any stimulation or spitting, and approximately 3 mL of saliva was collected from each subject. Using a micropipette, saliva was drawn from a disposable cup, transferred to a sterile plain tube, centrifuged at 4000 rpm for 3 min, placed into an uncontaminated Eppendorf tube, ^28^ and then frozen at -20 °C immediately before ELISA analysis for citH3, ELA, CALPRO, and MPO biomarkers.

### Screening of NETosis biomarkers in saliva

Using manual ELISA technology, the levels of citH3 (EPIGEN TEK), ELA, CALPRO, and MPO (Cloud-Clone Corp) biomarkers in saliva were determined for each participant.

### Clinical periodontal parameters

Following saliva sampling, the clinical parameters (PLI, BOP, PPD, and CAL) and periodontal status was determined using William’s periodontal probe. Six distinct areas of each tooth (Distobuccal, Buccal, Mesiobuccal, Distolingual, Lingual, and Mesiolingual) were inspected to perform clinical parameters BOP/PPD and CAL only, while the scores of PLI were recorded by examining only four surfaces (mesial, distal, labial/buccal, lingual/palatal). The 3 ^rd^ molar was excluded from the examination of all parameters, except PLI. PLI was recorded using disclosing agents to detect the presence or absence of dental plaque, ^29^ while the percentage of BOP was noted as 1 present 0 absent. ^30^ The distance from the free gingival margin to the base of the pocket is known as PPD, whereas the distance from the cemento-enamel junction (CEJ) to the base of the pocket in the absence of gingival recession is called CAL. If the margin of the gingiva is at the CEJ, then the CAL was considered to be equal the PPD; when there is gingival recession, CAL was determined by adding the recession depth and PPD; and when the margin was higher than CEJ, in order to determine the CAL, the distance from gingival margin to CEJ was subtracted from the PPD. ^25^ Staging was performed by calculating the CAL plus 2 mm of biological width per root length multiplied by 100 for the most affected tooth in the dentition.

### Calibration

The calibrated periodontal evaluations were completed and performed on five patients not included in the study at the Periodontics Department/Dentistry College/Baghdad. The kappa-coefficient assay was used to examine categorical variables to prevent false-positive results. Clinical periodontal data (PPD and CAL) were recorded every two hours for each of the six sites per tooth (except for the third molars). Measurement reliability was confirmed by the coefficients of intra-class correlation (ICC) of 0.950 for PPD and 0.860 for CAL, while those for PLI and BOP were 0.917 and 0.890, respectively.

### Statistical analysis

Data description, analysis, and presentation were performed using the Statistical Package for Social Sciences (SPSS) (version -22/Chicago/Illinois/USA) ( https://research-repository.uwa.edu.au/en/publications/spss-statistics-version-22-a-practical-guide ). ^31^ For quantitative variables, the descriptive analysis produced the mean and standard deviation (SD), while the percentage and frequency were qualitative variables. The kappa statistical test was employed to evaluate the two readings for dichotomous variables. The linear connection between the two quantitative variables was assessed using the Pearson’s correlation parametric test. Normality of distribution was verified using the Shapiro-Wilk test. Homogeneity was verified using Levene’s test. Two independent sample T test were used to determine how the two groups differed from one another. Two qualitative variables, distributional correlations, were tested using the chi-square test. Receiving operating characteristic curves (ROC) were used to calculate the sensitivity and specificity of each salivary biomarker.

### Pilot study

Ten participants from the dental teaching hospital at the Dentistry College/Baghdad University participated in a pilot study to investigate the association between NETosis biomarkers (citH3, ELA, CALPRO, and MPO) as indicators of an inflammatory response in smokers and nonsmokers with periodontitis.

## Results

**Figure 1.**
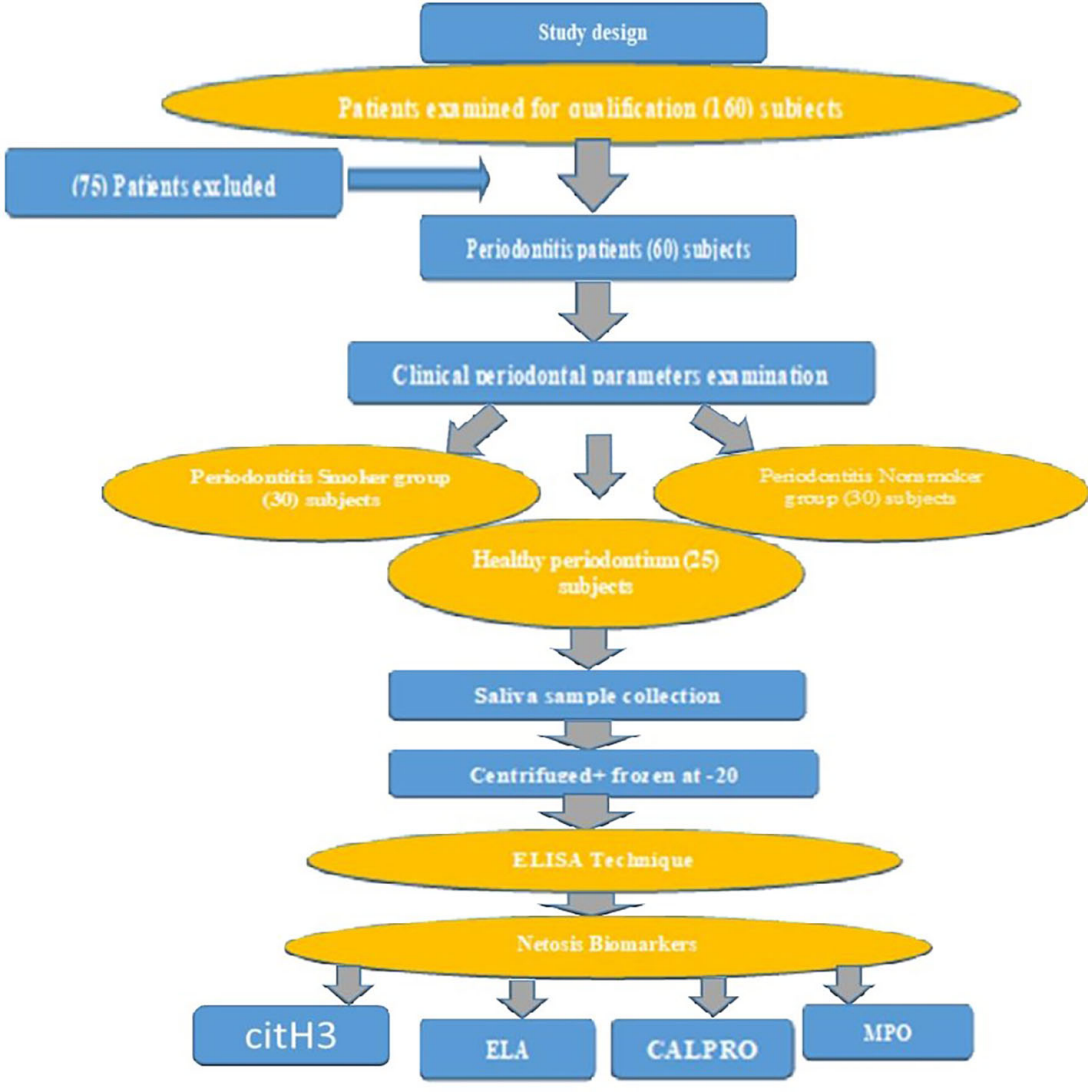
Flow Chart of the study design.

A total of 150 patients with periodontitis were screened to evaluate their eligibility for recruitment, and 75 people were excluded from the study after applying the inclusion and exclusion criteria to patients undergoing periodontal care clinics. Ultimately, only 60 male patients with periodontitis and 25 controls were included in the study ( Figure 1).

### Clinical periodontal parameters of the study groups

When comparing the periodontitis groups (smokers and nonsmokers) to the control group, the periodontal parameters of all 85 male study participants increased considerably ( Table 1). The mean values of the parameters (PLI/BOP/CAL) were compared among the study groups and showed a significant difference (p<0.05); smokers periodontitis patients had the highest mean levels of PLI and CAL, while the nonsmokers’ group had the highest elevation of the mean percentage of BOP. The smokers group also had a slightly higher mean PPD than the nonsmokers group; however, the results showed non-significant differences in both smokers and nonsmokers stage III and stage IV periodontitis groups.

**Table 1.** Mean of the clinical periodontal parameters of the study groups.

Clinical Parameters and age	Controls (n= 25)	Smokers periodontitis (SP)(n=30)	Nonsmoker periodontitis (NSP)(n=3)	Study groups P value	Multiple pairwise comparison (mean difference)					
					SP VS controls	P value	NSP Vs. controls	P value	SP vs. NSP	P value
Age										—
Mean ±SD	36.72±11.24	40.47±11.27	43.90±9.77	0.053 NS	—	—	—	—	—	
PLI										
Mean ±SD	22.58±10.03	60.84±27.58	54.11±24.59	*0.000	38.258	*0.000	31.530	*0.000	6.727	0.582NS
BOP										
Mean ±SD	5.28±2.41	34.29±20.08	42.29±18.31	*0.000	29.011	*0.000	37.016	*0.000	8.004	0.248 NS
PPD									—	—
Mean ±SD	—	0.4777±0.859	4.500±0.543	0.140 NS	—	—	—	—		
CAL					—				—	—
Mean ±SD	—	5.695±1.179	5.045±0.957	*0.022		—	—	—		

NS: non-significant, p>0.05;

* , significant p<0.05; n, number; PLI, plaque index; BOP, bleeding on probing; PPD, probing pocket depth; CAL, clinical attachment loss. SP: Smokers periodontitis, NSP: nonsmokers periodontitis.

**Figure 2.**
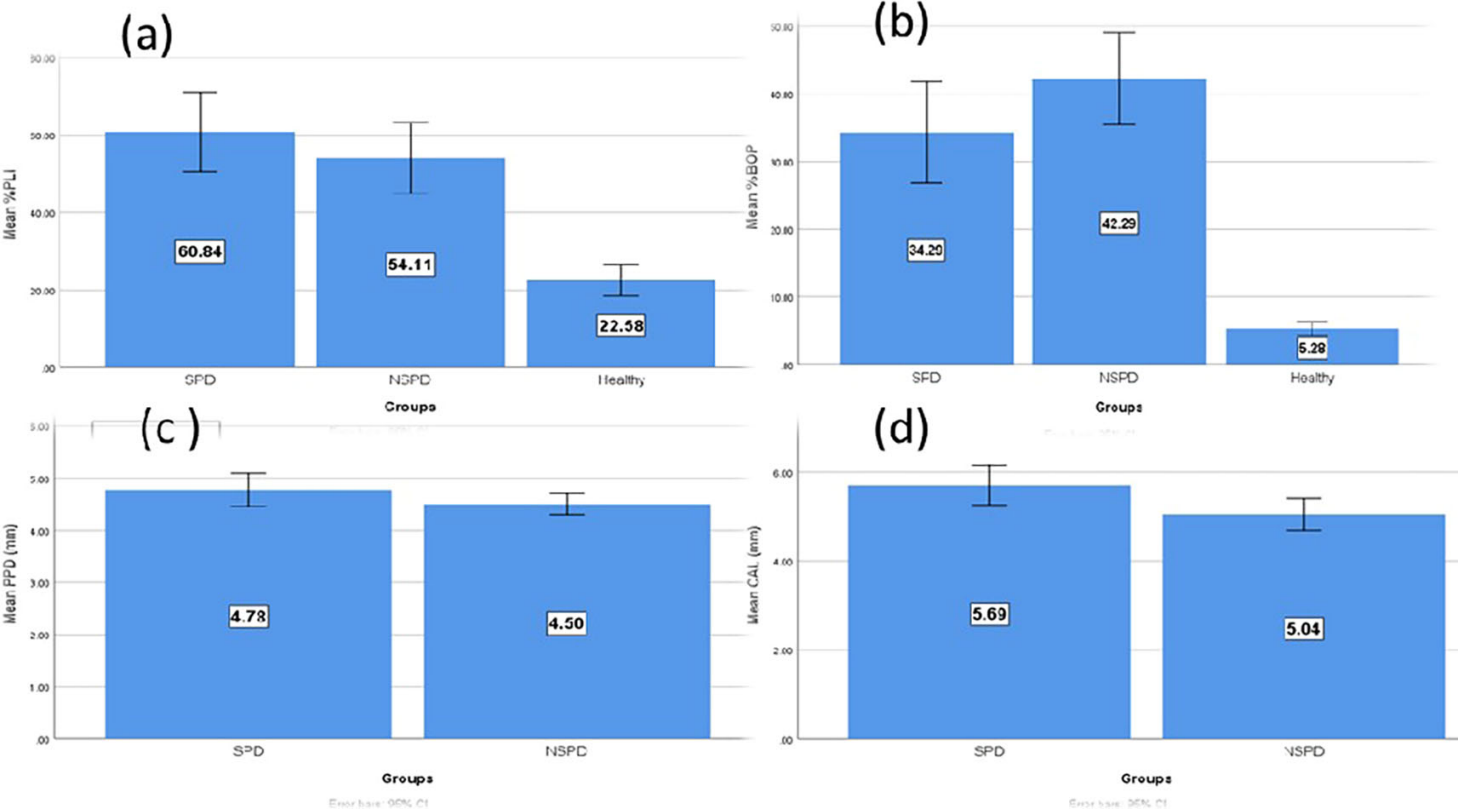
Clinical parameter comparison between research groups; (a) Mean of PLI, (b) Mean of BOP, (c) Mean of PPD, (d) Mean of CAL.

According to multiple pairwise comparisons, as shown in Table 1 between smokers and nonsmokers periodontitis groups, the difference in the average levels of PLI, BOP, and PPD was not statistically significant; however, there was a significant difference in the average CAL of smokers and nonsmokers with periodontitis. The difference in the mean levels of clinical periodontal parameters between the research groups is shown in Figure 2.

### Salivary biomarkers levels

Table 2 illustrates that the average levels of salivary biomarkers were higher in the periodontitis groups (smokers and nonsmokers) than in the controls, while the highest level was in the smokers group and the multiple pairwise comparison between the groups was statistically significant.

**Table 2.** Salivary biomarkers’ mean levels among groups.

Salivary biomarkers	Controls (n= 25)	Smokers periodontitis (SP) (n=30)	Nonsmoker periodontitis (NSP) (n=30)	Study groups P value	Multiple pairwise comparison (mean difference)					
					SP Vs. controls	P value	NSP Vs. controls	P value	SP Vs. NSP	P value
citH3 Mean ± SD	0.49 ± 0.59	2.95 ± 0.64	2.24 ± 0.55	*0.000	2.533	*0.000	1.818	*0.000	0.715	*0.000
ELA Mean ± SD	0.63 ± 0.18 Β	3.12 ± 0.81	1.83 ± 0.45	*0.000	2.488	*0.000	1.193	*0.000	1.295	*0.000
CALPRO Mean ± SD	153.12 ± 93.38	382.96±88.50	308.36 ± 117.23	*0.000	229.839	*0.000	155.244	*0.000	74.595	*0.016
MPO Mean ± SD	93.68 ± 22.0	369.06 ± 92.49	302.67 ± 94.23	*0.000	275.382	*0.000	208.995	*0.000	66.386	*0.021

* Significant, p<0.05;

n, number; SD, standard deviation; citH3, citrullinated histone H3; ELA, neutrophil elastase; CALPRO, calprotectin; MPO, myeloperoxidase.

**Figure 3.**
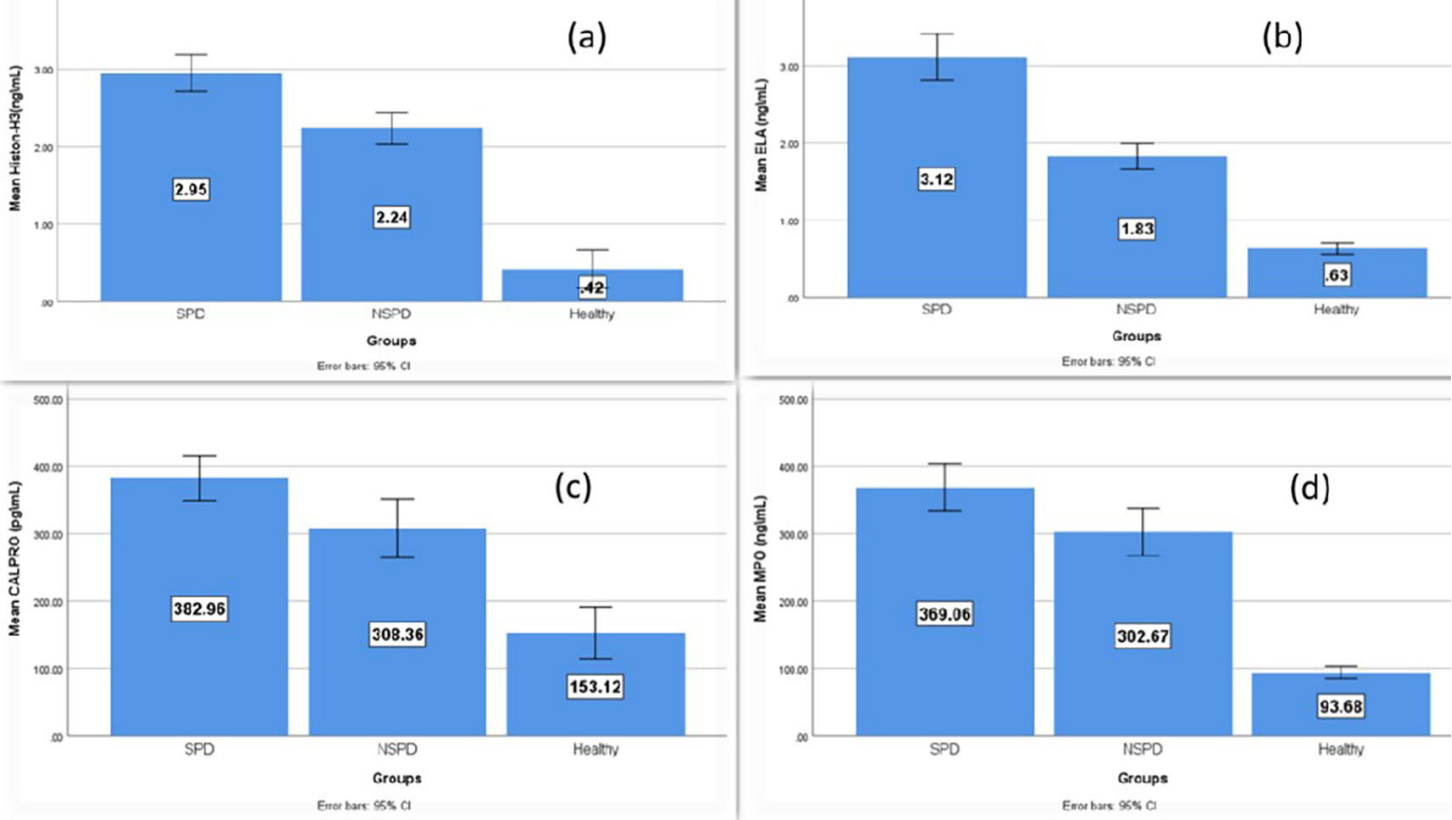
Comparison of the salivary biomarker among smokers’ periodontitis, nonsmokers’ periodontitis and controls, (a) citH3, (b) ELA, (c) CALPRO and (d) MPO.

Figure 3 demonstrates the comparison of the mean level of the NETosis salivary biomarkers (citH3, ELA, CALPRO, and MPO) among the study groups and that the higher elevation is in the smokers’ periodontitis group than in nonsmokers’ periodontitis and controls.

Table 3 demonstrates that the mean salivary biomarker levels in individuals with stage III and IV periodontitis showed a non-significant difference, regardless of whether they smoked.

**Table 3.** Salivary biomarkers’ mean levels among stage III and IV smokers and nonsmokers.

Salivary biomarkers	Periodontitis groups					
	Smokers stage III	Smokers stage IV	Nonsmokers stage III	Nonsmokers stage IV	P value smokers	P value nonsmokers
No.	22	8	20	10	—	—
citH3 Mean ± SD	2.88 ± 0.49	3.15 ± 0.95	2.27 ± 0.66	2.16 ± 0.24	0.313NS	0.611NS
ELA Mean ± SD	3.02 ± 0.79	3.40 ± 0.84	1.89 ± 0.49	1.70 ± 0.47	0.266NS	0.296NS
CALPRO Mean ± SD	393.65 ± 83.07	353.55 ± 101.96	305.26 ± 100.50	314.57 ± 151.30	0.280NS	0.842NS
MPO Mean ± SD	378.51 ± 89. 94	343.06 ± 100.59	289.57 ± 78.75	328.87 ± 119.91	0.362NS	0.289NS

NO: number, NS: non- significant, SD: standard deviation.

According to the comparison between periodontitis smokers and nonsmokers, as illustrated in Table 4, the mean salivary biomarker levels showed a non-significant association (p>0.05) between stage III and stage IV.

**Table 4.** Mean of the salivary biomarkers between stage Ill and IV of smokers and nonsmokers.

Group	Stage	No.	citH3 Mean ±SD	T test	P Value	ELA Mean ±SD	T test	P value	CALPRO Mean ±SD	T test	P value	MPO Mean ±SD	T test	P value
Smokers Periodontitis	III	22	2.88 ± 0.49	1.028	0.313 NS	3.02 ± 0.79	1.136	0.266	393.65 ± 83.07	1.101	.280 NS	378.51 ± 89.94	0.926	0.362 NS
	IV	8	3.15 ± 0.95			3.40 ± 0.84			353.55 ± 101.96			343.06 ± 100.59		
Nonsmokers Periodontitis	III	20	2.27 ± 0.66	0.515	0.611 NS	1.89 ± 0.44	1.066	0.296	305.26 ± 100.50	0.201	.842 NS	289.57 ± 78.75	1.080	0.289 NS
	IV	10	2.16 ± 0.24			1.70 ± 0.47			314.57 ± 151.30			328.87 ± 119.91		
Total	III	42	2.59 ± 0.65	0.053	0.962 NS	2.48 ± 0.86	0.098	0.922 NS	351.56 ± 101.05	0.634	.529 NS	336.16 ± 95.07	0.035	0.972 NS
	IV	18	2.60 ± 0.81			2.45 ± 1.08			331.89 ± 129.61			335.18 ± 108.77		

**Figure 4.**
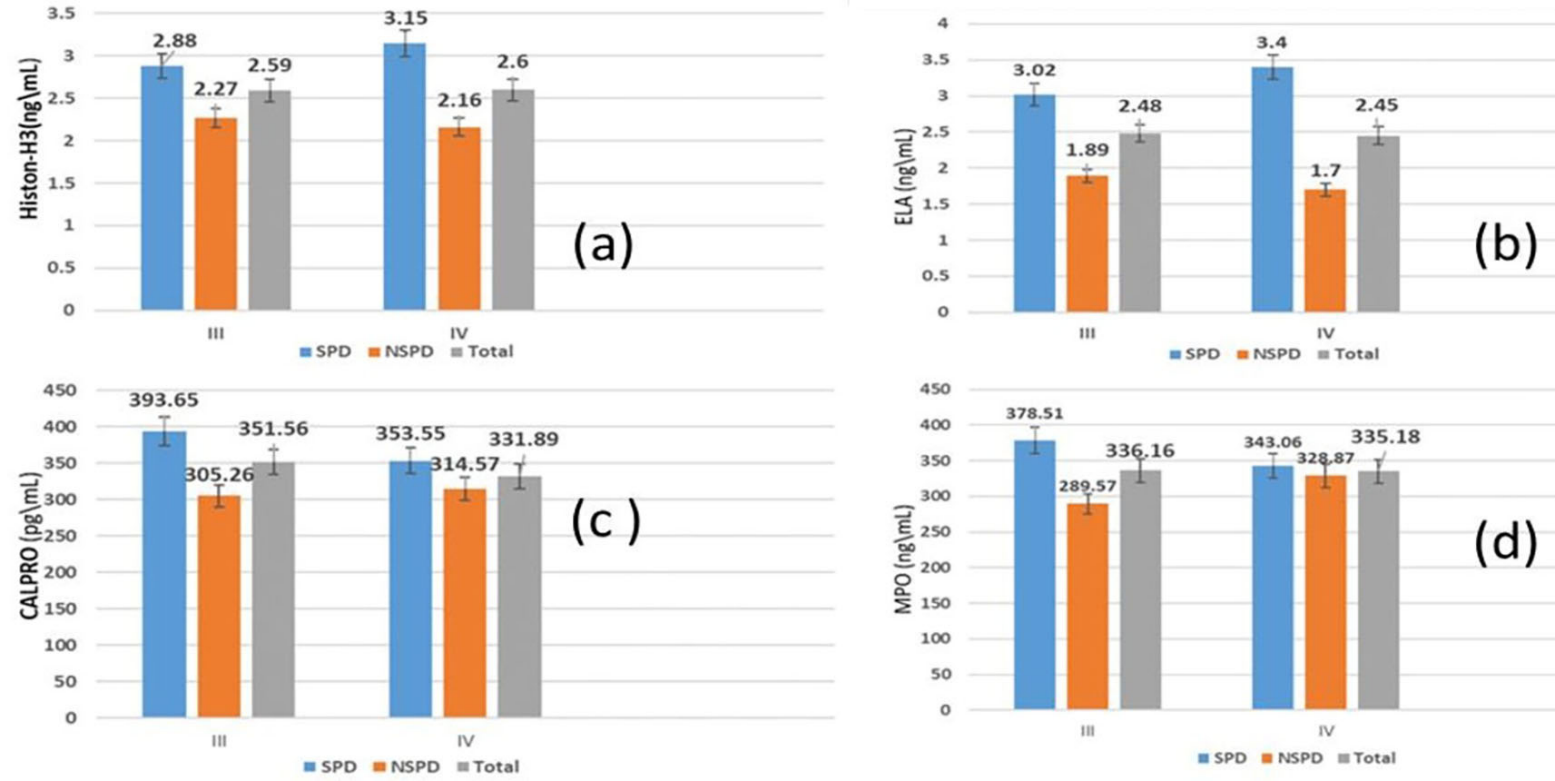
Comparison of Salivary biomarker between stage III and IV of periodontitis; (a) citrullinated histone H3 (citH3), (b) neutrophil elastase (ELA), (c) calprotectin (CALPRO), (d) myeloperoxidase (MPO).

On the other hand ( Figure 4), demonstrates in graphs the comparison of the mean of salivary biomarkers between stage III and IV periodontitis of both smokers and nonsmokers groups.

### Correlation between stage III and stage IV periodontal parameters with the salivary biomarkers

Regarding the Pearson Correlation Coefficient between stage III and IV periodontal parameters and the salivary biomarkers, there was a significant relationship (p<0.05) in stage III periodontitis between citH3 and CAL as well as between CALPRO and each of (PLI, PPD, and CAL), whereas in stage IV periodontitis, the correlation was significant only between MPO and CAL, as shown in Table 5.

**Table 5.** Correlation between stage III and IV periodontal parameters and salivary biomarker.

Stage		Histon-H3		ELA		CALPRO		MPO	
		r	p	r	P	r	P	r	p
III	PLI	-0.211	0.179	-0.003	0.985	0.340	*0.027	0.017	0.915
	BOP	-0.156	0.325	-0.051	0.750	0.219	*0.164	-0.157	0.319
	PPD	0.127	0.422	0.210	0.182	0.327	*0.015	0.073	0.644
	CAL	0.386	*0.012	0.178	0.260	0.312	*0.044	0.176	0.264
IV	PLI	0.333	0.177	0.378	0.122	0.074	0.771	0.264	0.289
	BOP	-0.288	0.246	-0.114	0.653	0.220	0.379	0.057	0.821
	PPD	0.346	0.159	0.417	0.085	-0.044	0.862	0.261	0.296
	CAL	0.030	0.906	0.100	0.693	0.392	0.108	0.570	*0.013

* Significant difference (p<0.05); r: coefficient correlation between -1 and 1; p: probability.

### Correlation among salivary NETosis biomarkers

Concerning the correlations between citH3 and CALPRO levels, a strong correlation was observed in the group of smokers with periodontitis, whereas the correlation of citH3 with ELA was strong in the group of nonsmokers with periodontitis, as illustrated in Table 6.

**Table 6.** Correlation among the salivary biomarkers.

Groups		ELA		CALPRO		MPO	
		r	p	r	p	r	p
SP	citH3	0.124	0.515	0.685	0.000	-0.034	0.857
	ELA			-0.266	0.156	0.231	0.219
	CALPRO			1		-0.084	0.657
NSP	citH3	0.535	0.002	-0.106	0.576	-0.027	0.887
	ELA			0.094	0.620	0.139	0.464
	CALPRO					0.215	0.254

### Relationship between biomarkers and clinical parameters

Based on the relationship between salivary biomarkers and clinical parameters, this study found a strong association between BOP% and CALPRO in smokers with periodontitis. Moreover, there was a strong association between BOP and ELA, as well as between CAL and MPO in the nonsmokers periodontitis group, as shown in Table 7.

**Table 7.** Correlation between periodontal parameters and salivary biomarkers.

Clinical Parameters of Groups		citH3		ELA		CALPRO		MPO	
		r	p	r	P	r	p	r	p
Smokers Periodontitis	PLI%	-0.068	0.720	0.013	0.944	0.165	0.384	-0.121	0.524
	BOP%	-0.341	0.066	-0.075	0.694	0.505	*0.004	-0.052	0.785
	PPD	0.201	0.288	0.230	0.220	-0.010	0.959	-0.052	0.785
	CAL	0.032	0.868	-0.215	0.255	0.097	0.608	0.057	0.765
Nonsmokers periodontitis	PLI%	0.136	0.472	0.129	0.495	0.219	0.244	0.254	0.176
	BOP%	0.175	0.356	0.459	*0.011	0.119	0.530	0.046	0.808
	PPD	0.005	0.981	0.002	0.992	-0.032	0.867	0.284	0.128
	CAL	0.076	0.689	0.005	0.980	0.226	0.230	0.379	*0.039

* Significant difference (p<0.05), r: correlation coefficient between -1 and 1, p: probability.

**Figure 5.**
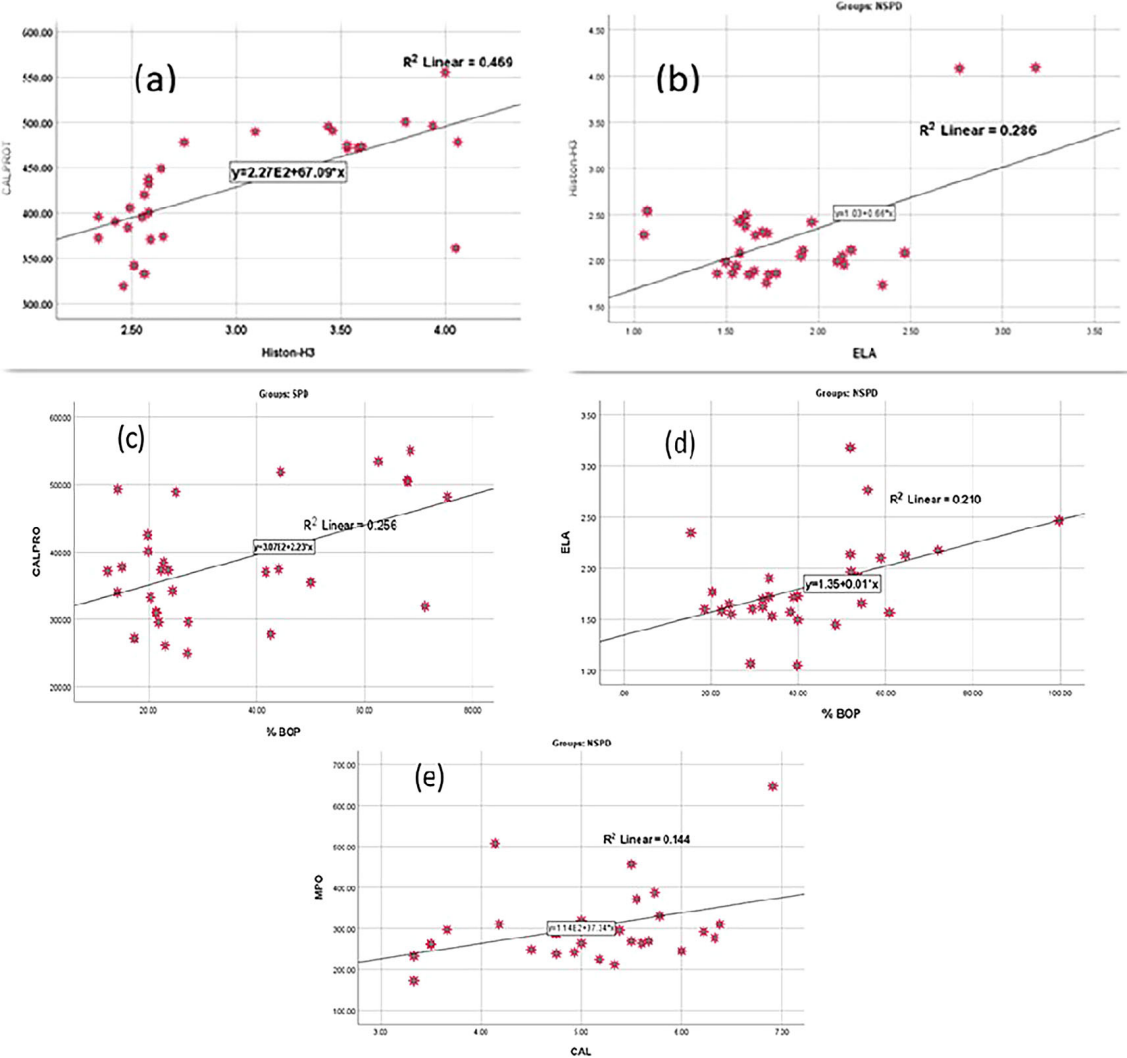
Correlation between; (a) CALPRO and citH3, (b) ELA and citH3, (c) CALPRO and BOP%, (d) ELA and BOP%, (e) MPO and CAL.

Figure 5 describes the linear correlation between citH3 and CALPRO levels in smokers periodontitis group, as well as between citH3 levels and ELA levels in nonsmokers with periodontitis. On the other hand, also describes the linear correlation between CALPRO and BOP%, ELA and BOP%, and MPO and CAL.

### Diagnostic accuracy of NETosis biomarkers

**Figure 6.**
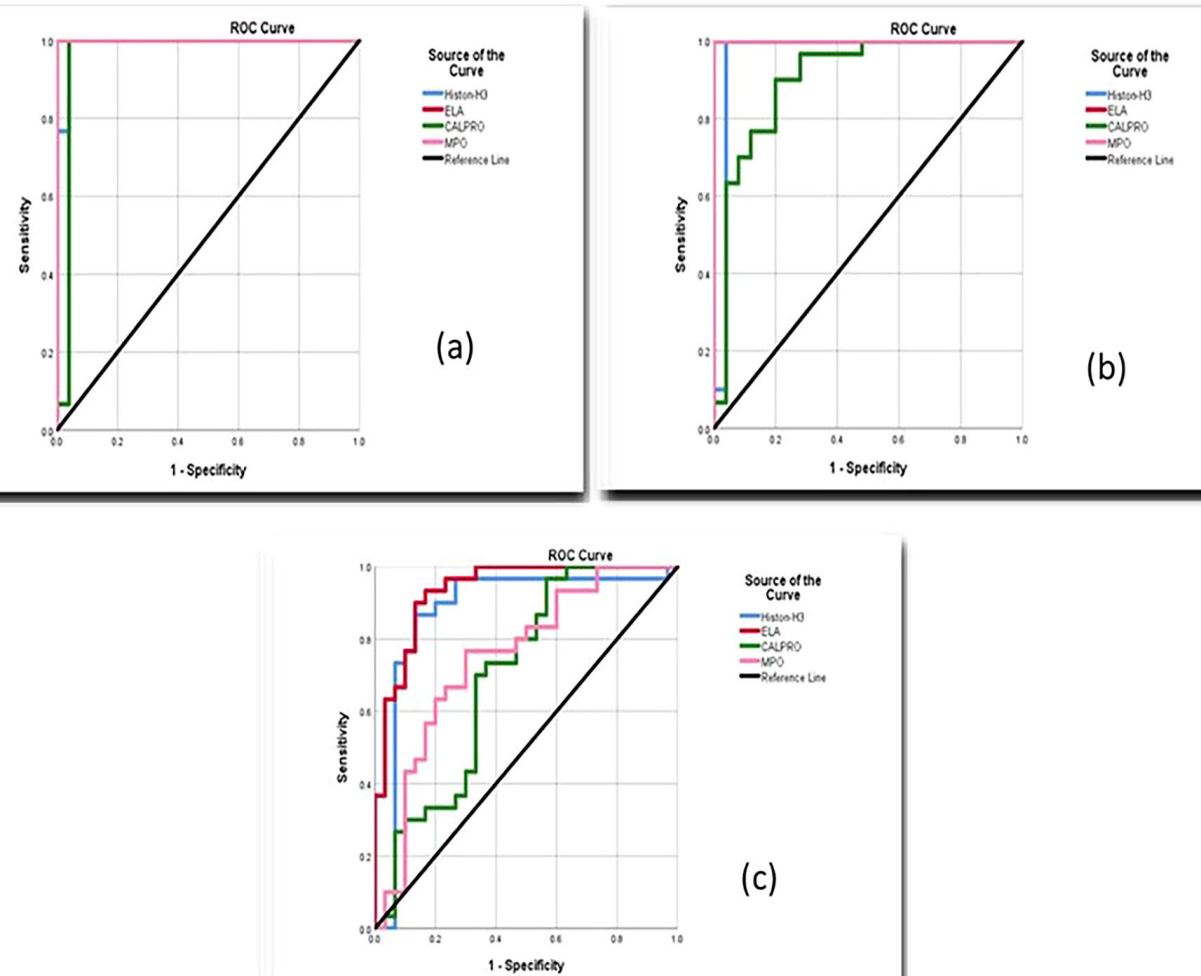
Receiver operating curves (ROC) for salivary biomarkers (a) Smokers periodontitis vs. control, (b) Nonsmokers periodontitis vs. control, (c) Smokers vs. nonsmokers.

Periodontitis smokers and nonsmokers, according to receiver operating curve (ROC) data, had good accuracy in estimating salivary biomarker sensitivity and specificity in differentiating periodontitis from healthy periodontium, as shown in Figure 6.

Additionally, the area under the curve (AUC) values between smokers periodontitis and control for salivary citH3, ELA, CALPRO, and MPO were 0.991-1,000, and the AUC between periodontitis nonsmokers and control for the selected markers was 0.964-1,000 also between smokers and nonsmokers periodontitis group was 0.878-0.750, as shown in Table 8.

**Table 8.** Comparison of the diagnostic properties of NETosis biomarkers among the study groups.

Comparison	Test result variables (s)	AUC	P value	Optimal cut of point	Sensitivity	Specificity
Smokers Periodontitis Vs. Controls	citH3	0.991	*0.000	2.041	96.7	96
	ELA	1.000	*0.000	1.43	100	100
	CALPRO	0.963	*0.000	266.67	93	96
	MPO	1.000	*0.000	62.008	100	100
Nonsmokers Periodontitis Vs. Controls	citH3	0.964	*0.000	1.804	93	96
	ELA	1.000	*0.000	1.004	100	100
	CALPRO	0.903	*0.000	163.920	93.3	72
	MPO	1.000	*0.000	62.008	100	100
Smokers periodontitis Vs. Nonsmokers Periodontitis	citH3	0.878	*0.000	2.472	83.3	86.7
	ELA	0.937	*0.000	2.158	93.3	83.3
	CALPRO	0.700	*0.008	348.468	60	66.7
	MPO	0.750	*0.001	304.025	70	70

* Significant difference, AUC: area under the curve.

## Discussion

This study evaluated NETosis biomarkers in smokers and nonsmokers with periodontitis. The benefit of our case-control study was the use of multiple salivary biomarkers (citH3, ELA, CALPRO, and MPO), thus distinguishing between the biomarkers with the greatest impact on periodontitis groups. There are different factors that may increase the severity of periodontitis, one of which is cigarettes smoking. ^2^^–^^4^ According to our findings, CALPRO was significantly elevated in the saliva of male smokers in the periodontitis group. Cardoso et al. ^32^ found that the male sex plays a role in CALPRO in predisposing individuals to periodontitis. The current finding is in agreement with Wei et al. ^23^ who attributed elevated CALPRO to the recruitment of neutrophils in the inflammatory periodontitis site, which acts as an innate immune defense, and also found that CALPRO is used as an antibacterial agent released highly from neutrophils in response to periodontal disorders. Brembach et al. ^33^ reported that nicotine induced changes in the release of ROS, which is one of the defense mechanisms of neutrophils against inflammation, in addition to NET formation. Smoking has an enormous effect on the host response. However, the present study was consistent with a previous study, ^33^ and showed an elevation of CALPRO in smokers. However, D’Haens et al. ^34^ showed that the salivary level of S100A protein, one of the CALPRO components, is decreased in individuals with inflammatory disease, which leads to a decline in the host’s oral defense. Herein, our data revealed that there is a higher elevation of ELA in smokers with periodontitis than in nonsmokers with periodontitis and controls; the part of our study of increasing ELA production in smokers with periodontitis group was consistent with the study of Özkaca et al. ^35^ Our study also agrees with another recent study by Tseng et al., ^36^ who found that ELA promotes the progression and pathogenesis of periodontal disease. Pauletto et al. ^37^ disagreed with our findings, and found that ELA showed no significant change in smokers’ periodontitis and the level of ELA is increased in nonsmokers with periodontitis; therefore, more studies are needed to focus on the level of ELA in smokers with periodontitis. The other biomarker identified in this study was MPO. The current study detected higher elevation of the level of MPO in smokers with periodontitis; the current results were consistent with those of Omer et al., ^38^ who found that compromised periodontal inflammation in smokers may have increased the expression of MPO. Gattani et al. ^39^ also showed the same increase in MPO level in smokers with periodontitis than in nonsmokers with periodontitis, as it is considered as an indicator of oxidative stress caused by cigarette smoke and increased level of inflammation in the progression of periodontitis. However, a pilot study conducted by Magán-Fernández et al. ^40^ discovered that, without statistical significance, MPO expression was greater in periodontitis than in gingivitis. Additionally, this study found a significant elevation in citH3 levels in smoker with periodontitis compared with nonsmokers. This was consistent with a study by Okamato et al. ^20^ which found that citH3 is increased in individuals at risk than in control subjects. Kim et al. ^9^ agreed with our findings of histone elevation, which is highly associated with the presence of NETs and bone destruction, and that citH3 bears NET-associated posttranslational modification. Few studies have examined the effect of smoking on citH3 in periodontitis patients. A study performed by Hosseinzadeh et al. ^41^ demonstrated that smoking-related diseases may be aggravated by nicotine in cigarette smoke, which then activates the production of NETs. In contrast, Magán-Fernández et al. ^40^ reported that the expression of citH3 is higher in gingivitis than in periodontitis. Furthermore, the present study showed that the biomarkers were unable to differentiate between stage III and IV periodontitis groups. However, another recent study conducted by Mohammed et al. ^42^ agreed with our finding that the concentrations of salivary biomarkers matrix metalloproteinase (MMP8 and 9) and tissue inhibitor of metalloproteinase (TIMP1) were not significantly different (p<0.05) among the various periodontitis stages. Interestingly, the present study noticed a strong correlation between CALPRO and most of the clinical periodontal parameters in stage III periodontitis, thus enhancing the hypothesis that this biomarker performs an essential function in the NETosis stimulation process of periodontal inflammation, the present study finding was in line with Wei et al., ^23^ who found that CALPRO is positively correlated with clinical periodontal parameters in generalized aggressive periodontitis, and that there is a positive correlation between citH3 and CAL in stage III periodontitis, which is consistent with the findings of Kim et al., ^9^ who found that citH3 is strongly associated with bone destruction. Moreover, MPO showed a positive correlation with CAL. Similarly, Polizzi et al. ^24^ showed that generalized chronic periodontal disease is positively correlated with salivary MPO. There was a strong and positive association between citH3 and CALPRO in smokers with periodontitis. Our data also revealed a highly significant positive relationship between citH3 and ELA in nonsmokers’ periodontitis. The results of this study indicate that the elevation of citH3 in both periodontitis groups is indicative of the process of NETosis and increases inflammatory reactions, even if periodontitis risk factors are present, which emphasizes a possible interaction between neutrophil loss and the prevalence of autoimmune diseases. Elevation of these biomarkers in periodontitis smokers and nonsmokers aids in the external breakdown of pathogens that get stuck within NETs. A limitation of the current study is the high exclusion criteria proposed in the study. However, it provides additional specifications for our data. The novelty of this study is the limitation of studies that evaluated the effect of the citH3 NETosis salivary biomarker in smokers with periodontitis.

## Conclusions

The selected salivary biomarkers (citH3, ELA, CALPRO, and MPO) were used to identify differences between healthy periodontium and periodontitis. All the chosen biomarkers were elevated in the smoker periodontitis group, and there was a statistically significant difference between smokers and nonsmokers with periodontitis compared to the controls. Smoking appears to have harmful effects, one of which is the downregulation of immunological responses to bacterial infection. On the other hand, it reduces the ability of neutrophils to respond to periodontal infection; therefore, smoking increases the biomarker levels in the smokers periodontitis group. The citH3, ELA, CALPRO, and MPO levels could not differentiate between stage III and IV smokers and nonsmoker periodontitis groups. There was a significant correlation between the salivary biomarkers citH3 and CALPRO in the periodontitis group and between citH3 and ELA in the nonsmoker periodontitis groups. Interestingly, the salivary biomarkers citH3, ELA, and CALPRO were positively correlated. All the selected salivary biomarkers increased in cases of periodontitis and were associated with neutrophil enzymes that mediate NETosis and are connected to the onset and progression of periodontitis.

## Author’s contributions

Conceptualization Ideas: all authors RAN and BHA designed and performed the manuscript. RAN collected the patients, samples, and provided clinical information. RAN, BHA, MG, and AAA performed the experiments and contributed to the statistical analysis, reviewing the work for clarity and originality. RAN, BHA, and supervision by BHA. The authors reviewed and provided their stamp of approval for the paper. All necessary conditions for authorship have been fulfilled, and all the authors agree that the manuscript conveys their honest work.

## Consent to participate

Following a sequential presentation of the research purpose and methods, all participants signed an informed consent form. The samples that collected in this study were salivary samples and this study was conducted at University of Baghdad/College of Dentistry/Department of Periodontics after ethical approval with reference Number: 844, project number: 844823 in 15-10-2023 was obtained, and a written consent was taken from the participant.

## Ethics and consent

Ethical considerations were essential during the conduct of this study, introduced by the World Medical Association’s Helsinki Declaration (WMA), after submission to the ethical committee at the Dentistry College/University of Baghdad to obtain ethical approval (Reference Number: 844, project number: 844823, date: 15-10-2023).

## Data Availability

Figshare: Evaluation of NETosis (Neutrophil Extracellular Traps) Biomarkers in Smokers and Nonsmokers Periodontitis Patients. https://doi.org/10.6084/m9.figshare.25997872.v2 ^43^ The project contains the following underlying data:
• 2=F1000=Data=Rasha Najim--InFormt.xlsx. Data are available under the terms of the Creative Commons Attribution 4.0 International license (CC-BY 4.0).
