# Supplementary material for: Evaluation of NETosis (Neutrophil Extracellular Traps) biomarkers in smokers and nonsmokers periodontitis patients
Source: F1000Res. 2025 Apr 7;13:907. Originally published 2024 Aug 8. [Version 2] doi: 10.12688/f1000research.152917.2 (PMC12715858; doi:10.12688/f1000research.152917.2)
Supplement: Peer review history

# Reviewer report: Reviewer response for version 2

**DOI:** 10.5256/f1000research.176674.r434266

**Published:** 2025-12-02

**Authors:** Zina Ali Daily

**Affiliations:** 1 University of Al-Ameed,Karbala, Karbala, Iraq

The work of this study is clear and given an information to the severity of periodontitis in smokers and nonsmokers. However, it is better to add a few words about the statistical method within the methods of the abstract and add the value of p in the results of the abstract for further clarification.

 Regarding the use manual ELISA technology to determine the levels of citH3, ELA, CALPRO, and MPO biomarkers in saliva but it was not clarifying how to obtain these levels, so it would be preferable to explain the processes of ELISA.

 It is also preferable to add a source to an existing paragraph within the article and explain the reason for that (The calibrated periodontal evaluations were performed on five patients which recorded every two hours for each of the six sites per tooth). Who did the examinations?

Is the work clearly and accurately presented and does it cite the current literature?

Yes

If applicable, is the statistical analysis and its interpretation appropriate?

Yes

Are all the source data underlying the results available to ensure full reproducibility?

Yes

Is the study design appropriate and is the work technically sound?

Yes

Are the conclusions drawn adequately supported by the results?

Partly

Are sufficient details of methods and analysis provided to allow replication by others?

Partly

Reviewer Expertise:

Immunology, genetics, periodontics, oncology, endocrine disorders, cardiovascular diseases and dental implants

I confirm that I have read this submission and believe that I have an appropriate level of expertise to confirm that it is of an acceptable scientific standard.

# Reviewer report: Reviewer response for version 2

**DOI:** 10.5256/f1000research.176674.r381719

**Published:** 2025-05-28

**Authors:** Mukhatar Ahmed Javali

**Affiliations:** 1 King Khalid University,, Abha, Saudi Arabia

Major Comments: Objective of the study is novel, the manuscript needs a proper english language for better understanding of what the authors are willing to prove by this study. Many sentences or paragrphs are not clear , to be refrased or rewritten.

 The objective to be clear and same in all parts of the study, whether its abstract,introduction, results, discussion or conclusion of the study. The objectives to be addressed as same.

 Plz see these >>>

 Abstract:

 Rewrite or better to write the sentence..****

**in the severe stages of periodontitis III and IV….as… In both smokers and nonsmokers stage III and IV periodontitis patients.**

 Moreover, the mean NETosis biomarker‘s mean levels were significantly higher in smoker than in nonsmokers….. **Refrase this sentence for better clarity**

 Smoking is one of the main ways in which individuals are exposed to harmful chemicals (cigarette smoke contains at least 4,500 different chemicals), and can cause epigenetic changes that increase inflammation. In addition, smoking generally increases oxidative stress, with excess free oxygen radicals causing cell damage…. **Cite a reference for this sentences**

 Several studies have indicated that smokers are more likely to have periodontal disease than nonsmokers, including greater gingival recession, **bone attachment**, and tooth loss…. **What does this mean**

 However, severe periodontitis is caused by a lack of neutrophils, and an excess of neutrophils appears to be harmful because it maintains the body in an inflammatory state, which destroys tissue….. Lack or defects in neutrophil or its functions… **Kindly refrase this sentence and provide a reference**

 Based on the **available evidence**, this study evaluated the NETosis biomarkers as indicators of inflammation in stage III and stage IV (smokers and nonsmokers) periodontitis groups and compared the findings with those of the controls. Furthermore, we determined the relationship between the NETosis biomarkers and clinical periodontal parameters….. Evidence is already available…. **Then what was the idea behind this study … to prove what is already evident…. Better to rewrite the aim or objectives of the study.**

 The type of this study was case control study not clinical trial study… W **hat the need to write this here… when already in above para the study design is explained.**

 Study population: Follow the case definition criteria given in the new classification 2017 for periodontitis and implant disease for staging or grading of periodontitis.

 Inclusion and exclusion criteria to be rewritten… more criteria’s are overlapping among inclusion and exclusion. And the study is done in smokers…. **But history of smoking is written as exclusion criteria.**

 Following saliva sampling, the clinical parameters (PLI, BOP, PPD, and CAL) and periodontal status was determined using William’s periodontal probe. Six distinct areas of each tooth (Distobuccal, Buccal, Mesiobuccal, Distolingual, Lingual, and Mesiolingual) were inspected to perform clinical parameters BOP/PPD and CAL only, while the scores of PLI were recorded by examining only four surfaces (mesial, distal, labial/buccal, lingual/palatal). The 3rd molar was excluded from the examination of all parameters, except PLI. PLI was recorded using disclosing agents to detect the presence or absence of dental plaque,29 while the percentage of BOP was noted as 1 present 0 absent.30 The distance from the free gingival margin to the base of the pocket is known as PPD, whereas the distance from the cemento-enamel junction (CEJ) to the base of the pocket in the absence of gingival recession is called CAL. If the margin of the gingiva is at the CEJ, then the CAL was considered to be equal the PPD; when there is gingival recession, CAL was determined by adding the recession depth and PPD; and when the margin was higher than CEJ, in order to determine the CAL, the distance from gingival margin to CEJ was subtracted from the PPD.25 Staging was performed by calculating the CAL plus 2 mm of biological width per root length multiplied by 100 for the most affected tooth in the dentition…. **This whole para is confusing… make it simple understandable**

 Pilot study

 Ten participants from the dental teaching hospital at the Dentistry College/Baghdad University participated in a pilot study to investigate the association between NETosis biomarkers (citH3, ELA, CALPRO, and MPO) as indicators of an inflammatory response in smokers and nonsmokers with periodontitis…… **Is it necessary to include these… What is the idea here to include**

 A total of 150 patients with periodontitis were screened to evaluate their eligibility for recruitment, and 75 people were excluded from the study after applying the inclusion and exclusion criteria to patients undergoing periodontal care clinics. Ultimately, only 60 male patients with periodontitis and 25 controls were included in the study (Figure 1)….. **Cross check the numbers and figures details.**

**The controls were taken from these 150 or 160 patients? What is control group…. Periodontal healthy individuals?**

**Saliva collection method to be given in details and be clear,**

**Dont repeate the tables or figures or graphs details in the results.**

**Include a para for limitaions of your study and future recommendations.**

**Conclusion part to have some recommendations at the end and some points regarding clinical relevance to the finding of the study.**

Is the work clearly and accurately presented and does it cite the current literature?

Partly

If applicable, is the statistical analysis and its interpretation appropriate?

I cannot comment. A qualified statistician is required.

Are all the source data underlying the results available to ensure full reproducibility?

Partly

Is the study design appropriate and is the work technically sound?

Partly

Are the conclusions drawn adequately supported by the results?

Partly

Are sufficient details of methods and analysis provided to allow replication by others?

Partly

Reviewer Expertise:

NA

I confirm that I have read this submission and believe that I have an appropriate level of expertise to confirm that it is of an acceptable scientific standard, however I have significant reservations, as outlined above.

# Reviewer report: Reviewer response for version 2

**DOI:** 10.5256/f1000research.176674.r376530

**Published:** 2025-05-19

**Authors:** Amal Bouziane

**Affiliations:** 1 Research Laboratory in Oral Biology and Biotechnology, Faculty of Dental Medicine,, Mohammed V University in Rabat, Rabat, Morocco; 2 Department of Periodontology, Faculty of Dental Medicine,, Mohammed V University in Rabat, Rabat, Morocco; 3 Laboratory of Biostatistics, Clinical Research and Epidemiology,, Mohammed V University in Rabat, Rabat, Morocco

The manuscript had many methodological and redactional flaws and required improvements recommended at the first submission.

 Several improvements have not been considered, including the title, Methods, and Results sections.

**​​​​​​1.    Title:**

 •    As NET is a production of NETosis, the abbreviation in the title should be reviewed.

 •    Please specify that periodontitis is a stage III and IV periodontitis.

**2.    Introduction:**

 ​​​​​​•    The sentence “ .. severe periodontitis is caused by a lack of neutrophils, and an excess of neutrophils appears to be harmful..” still needs clarification. 

 •    Text added to abbreviations for the first time is still needed. The list of Abbreviations at the beginning of the manuscript is not mandatory.​​​​​​

**​​​​​​3.    Methods:** 

 •    Please avoid redundancy in the study design. “..not clinical trial study” could be removed.

 •    The case definition of periodontitis, according to EFP and AAP 2017 consensus criteria, should be revised.

 •    “ any periodontal treatment” still need to be clarified.

 •    Smoking was a main exposition in the study. “History of smoking” could not be in exclusion criteria.

 •    In the paragraph “Clinical periodontal parameters, the criteria for staging is not common and need a reference (“ Staging was performed by calculating the CAL plus 2 mm of biological width per root length multiplied by 100 for the most affected tooth in the dentition.”) 

 •    Conducting the pilot study is still not justified in the methods.

 •    Please give a clear image of Flow Chart.

**4.    Results:**

 •    It is recommended to avoid redundancy between figures, tables and text for the same results

 •    0.000 should be replaced by <0.001 for the p value.

 •    Indicate the abbreviations under the tables 

 •    The results should be presented as a text narratively (example, the paragraph “Salivary biomarkers levels” ). 

 •    The terms used for the same meaning should be unified (example: relationship, correlation, association, on page 8)

 •    The accuracy of a diagnosis test needs a different method to state of Diagnostic accuracy of NETosis biomarkers. 

 •    Present the most relevant graphs.

 •    Present the most relevant results ( mean levels, AUC,..) of NETosis biomarkers, such as smokers Vs non-smokers.

Is the work clearly and accurately presented and does it cite the current literature?

Partly

If applicable, is the statistical analysis and its interpretation appropriate?

Partly

Are all the source data underlying the results available to ensure full reproducibility?

Yes

Is the study design appropriate and is the work technically sound?

Partly

Are the conclusions drawn adequately supported by the results?

Partly

Are sufficient details of methods and analysis provided to allow replication by others?

Yes

Reviewer Expertise:

NA

I confirm that I have read this submission and believe that I have an appropriate level of expertise to state that I do not consider it to be of an acceptable scientific standard, for reasons outlined above.

# Reviewer report: Reviewer response for version 2

**DOI:** 10.5256/f1000research.176674.r377248

**Published:** 2025-04-18

**Authors:** Gaetano Isola

**Affiliations:** 1 University of Catania, Catania, Italy

In the manuscript entitled: "Evaluation of NETosis (Neutrophil Extracellular Traps) biomarkers in smokers and nonsmokers periodontitis patients" the authors aimed to assess NETosis biomarkers citrullinated histone H3 (citH3), neutrophil elastase (ELA), calprotectin (CALPRO), and myeloperoxidase (MPO) as indicators of inflammation in the severe stages of periodontitis III and IV in both (smokers and nonsmokers) patients, and to determine the correlation between NETosis biomarkers and clinical periodontal parameters.

 The authors found that the mean NETosis biomarker‘s mean levels were significantly higher in smoker than in nonsmokers. In addition, the correlations were significant between CALPRO and CitH3 in smokers and between ELA and CitH3 in nonsmokers.

 The authors concluded that the chosen salivary biomarkers of NETosis revealed elevated clinical accuracy in differentiating the studied periodontitis groups (smokers and nonsmokers) from controls. In addition, cigarette smoking increases the risk of periodontitis, and neutrophils in smokers with periodontitis exhibited more susceptibility to form neutrophil extracellular traps when compared with nonsmokers.

**Major comments:**

 In general, the idea and innovation of this study regards the analysis of the link between peridontitis and alveolar bone loss mediated by mediators is interesting and novel because the role these aspects in medicine are validated but further studies on this topic could be an innovative issue in this field could be open a creative matter of debate in literature by adding new information. Moreover, there are few reports in the literature that studied this interesting topic with this kind of study design.

 The study was well conducted by the authors; However, there are some concerns to revise that are described below.

 The introduction section resumes the existing knowledge regarding the important factor linked with the relationship between periodontitis and systemic diseases.

 However, as the importance of the topic, the reviewer strongly recommends, before a further re-evaluation of the manuscript, to update the literature through read, discuss and must cites in the references with great attention all of those recent interesting articles, that helps the authors to better introduce and discuss the relationship between periodontitis peri-implant diseases and systemic diseases and how periodontal treatment could improve alveolar bone loss outcomes:

 1) Isola G,et al., 2025 (Ref 1) 

 2) Isola G,et al., 2025 (Ref 2) 

 The authors should be better specified, at the end of the introduction section, the rationale of the study and the aim of the study. In the central section, should better clarify inclusions and exclusions criteria of the selected sample.

 Please better state the results obtained in the abstract.

 The discussion section appears well organized with the relevant paper that support the conclusions, even if the authors should better discuss the relationship regarding the by periodontitis in and risk of oxidative stress evolution in periodontitis patients. The conclusion should reinforce in light of the discussions.

 In conclusion, I am sure that the authors are fine clinicians who achieve very nice results with their adopted protocol. However, this study, in my view does not in its current form satisfy a very high scientific requirement for indexing in this journal and requests a revision before a further re-evaluation of the manuscript.

**Minor Comments:**

 Abstract: Better formulate the abstract section by better describing the aim of the study

 Introduction: Please refer to major comments

 Discussion: Please add a specific sentence that clarifies the results obtained in the first part of the discussion

Is the work clearly and accurately presented and does it cite the current literature?

Partly

If applicable, is the statistical analysis and its interpretation appropriate?

Yes

Are all the source data underlying the results available to ensure full reproducibility?

Partly

Is the study design appropriate and is the work technically sound?

Partly

Are the conclusions drawn adequately supported by the results?

No

Are sufficient details of methods and analysis provided to allow replication by others?

Partly

Reviewer Expertise:

Periodontology

I confirm that I have read this submission and believe that I have an appropriate level of expertise to confirm that it is of an acceptable scientific standard, however I have significant reservations, as outlined above.

## References

[1] : Relationship between periodontitis and systemic diseases: A bibliometric and visual study. *Periodontol 2000* .2025; 10.1111/prd.12621 10.1111/prd.12621 39775963 PMC12842847

[2] : Effect of Nonsurgical Mechanical Debridement With or Without Chlorhexidine Formulations in the Treatment of Peri-Implant Mucositis. A Randomized Placebo-Controlled Clinical Trial. *Clin Oral Implants Res* .2025; 10.1111/clr.14405 10.1111/clr.14405 39865359 PMC12066909

# Reviewer report: Reviewer response for version 2

**DOI:** 10.5256/f1000research.176674.r377245

**Published:** 2025-04-15

**Authors:** Tarcília Aparecida Silva

**Affiliations:** 1 Universidade Federal de Minas Gerais, Belo Horizonte, State of Minas Gerais, Brazil

The manuscript entitled “Evaluation of NETosis (Neutrophil Extracellular Traps) biomarkers in smokers and nonsmokers periodontitis” aims to assess the salivary concentration of citrullinated histone H3 (citH3), neutrophil elastase (ELA), calprotectin (CALPRO), and myeloperoxidase (MPO) in smokers and nonsmokers’ individuals with periodontitis.

 The topic is relevant to both dental and medical research. Data from 85 patients were analyzed. The authors conducted a pilot study, sample size calculation and calibration, steps that strengthens the methodology. However, the study presents shortcomings that weaken its clarity and impact, making it unsuitable for indexing in its current form. I have provided criticisms and suggestions for improvement, as follows:

**Introduction: ** It is important to clearly state how this study scientifically advance s in relation to previous literature.

**Introduction:** ‘However, severe periodontitis is caused by a lack of neutrophils, and an excess of neutrophils appears to be harmful because it maintains the body in an inflammatory state, which destroys tissue’. Please rephrase to improve clarity. It would be important to exemplify the conditions in which the lack of neutrophils/Nets results in worse PD e.g. Papillon-Lefévre syndrome.

**Methods:** Considering the potential influence of systemic conditions in the tested salivary biomarkers, it is important to inform how systemic diseases were excluded in this study population. The same concern exists about the medication use, please clearly state how these potential influences were managed.

**Methods:** ‘The patients were advised to clean and floss their teeth to maintain good oral hygiene’. It is not clear if it was a recommendation prior saliva sampling or if patients received instructions (how many sessions) before inclusion in the study.

**Methods:** For how long the saliva samples were stored at -20 ^o^C until ELISA? I advise to report it because it is not the ideal temperature (-80 ^o^C).

**Methods:** Please revise the inclusion/exclusion criteria. Also, include smoking as inclusion criteria for one of groups and detail the criteria e.g. type of cigarettes, etc.

**Methods:** Why only males were included? Vantages and limitations of this choice should be presented in the Discussion section.

**Methods:** Did authors observed differences in the salivary flow between the groups? Please discuss how could this impact the study´s findings considering the effect of smoking in the salivary flow.

**Results:** Data presentation is repetitive. There is no need to represent same results in Tables and Figures. I advise the authors to chose just one presentation for each dataset and focus in the most relevant results. Additional information may be moved to supplementary material.

**Results:** Type and frequency and duration of smoking was evaluated? Maybe authors could use statistical tests to assess the potential influence this variable.

**Results:** Considering differences in the PD parameters in the group’s smoker x non-smoker, variables should be tested in a multivariate model to assess the weight of each one in the target biomarkers.

**Discussion:** The limitation topic should be expanded e.g. one time-point sampling considering the fluctuation of these molecules in saliva, only male included etc.

**Minor suggestions:**

**Methods:** Kits/reagents should be followed by detailed information of product and company (city, state, country).

**Methods:** For ELISA, please cite general details of method, e.g. duplicates were run; data were corrected by protein content or salivary flow, etc.

**Figure 1:** Consider to move it to the supplementary material.

 Modify ‘polymorph nuclear’ by ‘polymorphonuclear’

 Change ‘research groups’ by ‘groups’

Is the work clearly and accurately presented and does it cite the current literature?

Yes

If applicable, is the statistical analysis and its interpretation appropriate?

Partly

Are all the source data underlying the results available to ensure full reproducibility?

Yes

Is the study design appropriate and is the work technically sound?

Yes

Are the conclusions drawn adequately supported by the results?

Yes

Are sufficient details of methods and analysis provided to allow replication by others?

Yes

Reviewer Expertise:

Dentisty; Oral Pathology

I confirm that I have read this submission and believe that I have an appropriate level of expertise to confirm that it is of an acceptable scientific standard, however I have significant reservations, as outlined above.

# Reviewer report: Reviewer response for version 1

**DOI:** 10.5256/f1000research.167730.r328722

**Published:** 2024-10-30

**Authors:** Amal Bouziane

**Affiliations:** 1 Research Laboratory in Oral Biology and Biotechnology, Faculty of Dental Medicine,, Mohammed V University in Rabat, Rabat, Morocco; 2 Department of Periodontology, Faculty of Dental Medicine,, Mohammed V University in Rabat, Rabat, Morocco; 3 Laboratory of Biostatistics, Clinical Research and Epidemiology,, Mohammed V University in Rabat, Rabat, Morocco

The study tries to give some evidence about variations in salivary biomarkers’ (Neutrophil Extracellular Traps) levels in smokers and nonsmokers periodontitis patients. The comparison also concerned periodontally affected patients versus control subjects with a healthy periodontal status. Authors try to bring support to the clinical accuracy of NETosis salivary biomarkers to differentiate periodontitis groups (smokers and nonsmokers) from controls. However, the manuscript has many methodological and redactional flaws and requires improvement. The main issue is related to the design of the study and the presentation of the results.

1. **Title**:

- As NET is a production of NETosis, the abbreviation in the title should be reviewed.
- Please precise that periodontitis is a stage III and IV periodontitis.

1. **Introduction**:

- Physiopathology and risk factors of periodontitis need more clarification. Genetics should not be considered as a local factor.
- The sentence “ .. *severe periodontitis is caused by a lack of neutrophils, and an excess of neutrophils appears to be harmful*..” seems to be contradictory. Idem for “.. *The primary defense of periodontal tissues is provided by NETs, and a deficiency in NETs can result in severe early-onset periodontitis. Smoking cigarettes accelerates the production of NETs and PMN-derived proteases, which are responsible for breaking down connective tissue*..”. Please clarify.
- Please add text to abbreviations at the first time.

1. **Methods**:

- The main issue is related to the design of the study. It is not clear if case is Periodontitis group and control is Non periodontitis group, Or case is smokers group and control is Non smokers group. The outcome conditions the sample size and the selection of the study population. Periodontitis cases, including smokers and nonsmokers, should be compared with non-periodontitis controls including smokers and nonsmokers.
- Please avoid redundancy in the study design. “.. *not clinical trial study”* could be removed.
- Please review the case definition of periodontitis according to EFP and AAP 2017 consensus criteria.
- What do the authors mean by “ any periodontal treatment” in Exclusion criteria? Rarely, a patient did not receive a periodontal treatment in the past, at least a periodontal scaling.
- Smoking was a main exposition in the study. Smoking could not be an exclusion criterion.
- Please give a sharp Flow Chart, and add participants with healthy periodontal status.
- Why the pilot study was conducted? Please explain its part in the methods.

1. **Results**:

- The “increase” of parameters implies a prospective design study. Please give an appropriate word. Idem for elevation.
- The Statistical analysis “ *Two qualitative variables, distributional correlations, were tested using the chi-square test.*” Should be clarified.
- It is more appropriate to focus results to the objectives of the study. Figures 2 and 3 contain repeated information. They could be removed.
- 0.000 should be replaced by <0.001 for the p value.
- Indicate the abbreviations under the tables (for example table 5)
- Please begin the paragraph “Salivary biomarkers levels” with text.
- Present the most relevant graph in Figure 5.
- The first paragraph of ROC results should be reformulated. The accuracy of a diagnosis test needs a different method to state of Diagnostic accuracy of NETosis biomarkers.

1. **Discussion**:

- How the absence of correlation between CAL and Histon-H3, in stage IV, could be explained comparatively to stage III? (see Table 3)

Is the work clearly and accurately presented and does it cite the current literature?

Partly

If applicable, is the statistical analysis and its interpretation appropriate?

Partly

Are all the source data underlying the results available to ensure full reproducibility?

Yes

Is the study design appropriate and is the work technically sound?

Partly

Are the conclusions drawn adequately supported by the results?

Partly

Are sufficient details of methods and analysis provided to allow replication by others?

Yes

Reviewer Expertise:

NA

I confirm that I have read this submission and believe that I have an appropriate level of expertise to confirm that it is of an acceptable scientific standard, however I have significant reservations, as outlined above.

# Response

**Published:** 2025-01-06

**Authors:** Rasha Najim

**Affiliations:** Oral Microbiology, Basic Scien, University of Baghdad, Baghdad, Baghdad Governorate, Iraq

Greetings 

 Title 

 - I approved

 - NETosis was mentioned in the title to make the title more influential but we can omit it so I approve the new title ''Evaluation of neutrophil extracellular traps biomarkers as an indication in smokers and nonsmokers biomarkers stage III and IV periodontitis patients''

 Introduction

 - I approved

 - The clarifying for this sentence is that the balance that occurred in the number  neutrophils is crucial for maintaining periodontal homeostasis, Neutrophil equilibrium is important in periodontal homeostasis because periodontitis can develop when there are too many or too few neutrophils in the tissue.as an imbalance can result in defect in NETs production and then periodontitis.

 Also  smoking cigarettes has several potential detrimental effects on periodontal health which impaired defenses against plaque bacteria, and interference with oxidative stress processes. The primary defense of periodontal tissues is provided by NETs,  Neutrophils aid in homeostasis and defense by producing neutrophil extracellular taps (NETs). Smoking cigarettes accelerates the production of NETs , which are responsible for breaking down connective tissue.

 - I approved

 Methods

 - The control group is generally healthy persons and also healthy periodontium with BOP less than 10% and also CAL less than 3mm and no periodontal pocket depth less than 4mm and also non smokers any type of smoke.

 - I approved

 - The patient didn't do receive any antibiotic or periodontal treatment in the last 3 months before collecting the sample

 - We mean that we exclude those patients who smoke any other types of smoke like Vape hookah only we select the cigarette's smoke patients

 - The healthy periodontal status were added in the flow chart 

 - the pilot study was made to obtain the preliminary data for the primary outcome measure, in order to calculate the required sample size 

 Results 

 - Two qualitative variables were tested to calculate the sensitivity and specificity of each salivary biomarker.

 - I approved

 - I approved

# Reviewer report: Reviewer response for version 1

**DOI:** 10.5256/f1000research.167730.r315884

**Published:** 2024-09-09

**Authors:** Zerina Hadzic

**Affiliations:** 1 Sarajevo School of Science and Technology, Sarajevo, Federation of Bosnia and Herzegovina, Bosnia and Herzegovina

The text of the research article titled: Evaluation of NETosis (Neutrophil Extracellular Traps) biomarkers in smokers and nonsmokers periodontitis patients requires major correction. The abstract is not clear and it does not give all the important details for understanding the research article, especially in the methodology section. From the title, abstract, but also Introduction it is not clear that the authors aimed to determine the salivary parameters only in male patients. It is also not elaborated clearly why the female patients were excluded. The introduction does not give any inside in the other similar studies and the background for their examination in patients with periodontitis. The classification of the patients in the stage III and IV periodontitis groups is not correct. Stage III and stage IV in the segment of bone loss are radiologicaly with bone loss to mid-third of root or beyond, but are separated by the number of teeth lost, and with complexity of stage III being applicable to stage IV and additional complexity including masticatory disfunction, bite collapse, drifting, flaring and less then remaining 20 teeth (10 opposing pairs). [ ref 1 ]

 Also it is not clear if the patients consumed and antioxidants and if this was an exclusion criteria applied. In the saliva sampling section it is not clear: did the patients brush before the sampling? how were samples defrosted? Also the time frame of saliva sampling is not clear. The authors state: Salivary samples were conducted between 9 am and 12 pm. Is the 12 pm: noon  or 00:00, since by definition noon is neither AM or PM. Also, study included PLI in 3rd molars but excluded the 3rd molar for other clinical periodontal indices. Were the 3rd molars included in the determination of periodontitis stage?

 Conclusion is not written clearly and does not correspond to the precise results and study groups.

Is the work clearly and accurately presented and does it cite the current literature?

No

If applicable, is the statistical analysis and its interpretation appropriate?

I cannot comment. A qualified statistician is required.

Are all the source data underlying the results available to ensure full reproducibility?

Partly

Is the study design appropriate and is the work technically sound?

No

Are the conclusions drawn adequately supported by the results?

No

Are sufficient details of methods and analysis provided to allow replication by others?

No

Reviewer Expertise:

Periodontal disease, Salivary biomarkers, Oxidative stress, Inflammatory biomarkers, Biofilm.

I confirm that I have read this submission and believe that I have an appropriate level of expertise to state that I do not consider it to be of an acceptable scientific standard, for reasons outlined above.

## References

[1] : Staging and grading of periodontitis: Framework and proposal of a new classification and case definition. *J Periodontol* .2018;89 Suppl 1: 10.1002/JPER.18-0006 S159-S172 10.1002/JPER.18-0006 29926952

# Response

**Published:** 2024-09-13

**Authors:** Rasha Najim

**Affiliations:** Oral Microbiology, Basic Scien, University of Baghdad, Baghdad, Baghdad Governorate, Iraq

This research was approved for its title and content after submitting a protocol to the Council of the College of Dentistry at the university of Baghdad. the abstract of the research sheds light on the research in general but all the details are mentioned in the research in detail. All salivary samples in this research were taken from males exclusively and females were excluded from the beginning before starting the research to provide standardization of the  salivary samples on this study and also due to that hormonal fluctuations during  female's lifetime may provide an exaggerated inflammatory response to the clinical periodontal parameters and produce significant changes in the periodontium according to previous studies. All the periodontitis patients taken in this study were sever stages stage III and stage IV and we depend on the clinical periodontal parameters according to the mentioned ref. (46) that depend on calculating the CAL and presence of PPD and all the parameters were assessed by inter-examiner as well as intra-examiner calibration. All the salivary samples as mentioned in this research were collected from specialized governmental dental educational centers and their working hours are necessarily from morning until 3 pm therefore the samples were taken in the morning period and before any periodontal treatment for the purpose of accuracy in collecting salivary samples.
